# The effect of task modality and stimulus frequency in paced serial addition tests on functional brain activity

**DOI:** 10.1371/journal.pone.0194388

**Published:** 2018-03-15

**Authors:** Jeroen Gielen, Wietse Wiels, Jeroen Van Schependom, Jorne Laton, Wim Van Hecke, Paul M. Parizel, Marie Beatrice D’hooghe, Guy Nagels

**Affiliations:** 1 Department of Neurology, UZ Brussel, Centre for Neurosciences (C4N), Vrije Universiteit Brussel (VUB), Brussels, Belgium; 2 National MS Center Melsbroek, Melsbroek, Belgium; 3 Service d'Orthopédagogie Clinique, Faculté de Psychologie et des Sciences de l'Education, Université de Mons, Mons, Belgium; 4 Icometrix, Leuven, Belgium; 5 Department of Radiology, Antwerp University Hospital & University of Antwerp, Antwerp, Belgium; University of Akron, UNITED STATES

## Abstract

**Introduction:**

The paced serial addition test (PSAT) is regularly used to assess cognitive deficits in various neuropsychiatric conditions. Being a complex test, it reflects the status of multiple cognitive domains such as working memory, information processing speed and executive functioning. Two versions of the PSAT exist. One uses auditory stimuli through spoken numbers and is known as the PASAT, while the other one presents patients with visual stimuli and is called PVSAT. The PASAT is considered more frustrating by patients, and hence the visual version is usually preferred. Research has suggested that an interference might exist between patients’ verbal answers and the auditory presentation of stimuli. We therefore removed the verbal response in this study, and aimed to investigate differences in functional brain activity through functional magnetic resonance imaging.

**Methods:**

Fifteen healthy controls performed the two test versions inside an MRI scanner—switching between stimulus modality (auditory vs. visual) as well as inter-stimulus frequency (3s vs. 2s). We extracted 11 independent components from the data: attentional, visual, auditory, sensorimotor and default mode networks. We then performed statistical analyses of mean network activity within each component, as well as inter-network connectivity of each component pair during the different task types.

**Results:**

Unsurprisingly, we noted an effect of modality on activity in the visual and auditory components. However, we also describe bilateral frontoparietal, anterior cingulate and insular attentional network activity. An effect of frequency was noted only in the sensorimotor network. Effects were found on edges linking visual and auditory regions. Task modality influenced an attentional-sensorimotor connection, while stimulus frequency had an influence on sensorimotor-default mode connections.

**Conclusions:**

Scanner noise during functional MRI may interfere with brain activation—especially during tasks involving auditory pathways. The question whether to use PVSAT or PASAT for an fMRI study is, therefore, an important one. Specific effects of both modalities should be known to study designers. We conclude that both tests should not be considered interchangeable, as significant changes were brought to light during test performance in different modalities.

## Introduction

Both the visual and auditory variants of the paced serial addition test (PVSAT and PASAT, respectively) are used to assess neuropsychological deficits such as information processing speed, working memory, executive function, arithmetical ability, etc. [1,2]) in different neuropsychiatric conditions. It is an important aspect of the neuropsychological and cognitive evaluation of patients and healthy controls alike, and is therefore used in various clinical and research-related settings. For example, PSAT scores may alert the clinician to cognitive problems that may benefit from future rehabilitation or disease-modifying agents.

During the administration of PASAT/PVSAT, subjects are presented with a predefined—yet seemingly random—series of single digit numbers. In clinical practice, subjects are asked to sum up each digit with the previously presented digit. For example, if the series of numbers is ‘1’, ‘5’ and ‘3’, the correct responses are ‘6’ and ‘8’. The delay between subsequent stimuli is usually set to either two or three seconds, a shorter interval resulting in a more difficult test.

Behavioural research has suggested that PASAT and PVSAT results may be interchangeable. Even though the auditory version (PASAT) is generally considered more frustrating by test subjects [3], this version is nevertheless used most frequently in clinical practice. It is, for example, included in a widely used neuropsychological test battery to assess cognitive deterioration in multiple sclerosis (MS) (Multiple Sclerosis Functional Composite, MSFC, [4]). Interestingly, one behavioural study suggested that both tests are interchangeable by showing high correlations (r > 0.7, p < 0.001) between both versions–even when using different delays both in healthy controls and MS patients [5].

Several explanations have been suggested as to why the PASAT is perceived as less enjoyable than the PVSAT. The most plausible suggestion is the existence of an interference between vocalizing the answers while listening to the next stimulus. Tombaugh et al. suggested that lower performance during PASAT was the result of an interference effect where both stimulus input and response are processed through a single auditory channel [6], which is not the case in PVSA Testing. Additionally, scanner noise during functional MRI measurements may interfere with brain activation, especially during tasks that involve the auditory pathways [7]

The aim of this study was to investigate which brain networks are activated by paced serial addition testing during functional MRI (fMRI) registration, and how these activations change during different versions of the test, in a group of healthy volunteers.

## Methods

### Subjects

Fifteen healthy controls were recruited. All were female students enrolled in the Master of Biomedical Sciences at the University of Antwerp (UA). The study was approved by the UA ethical committee and all subjects gave their written informed consent.

### Acquisition

Functional MR images were collected using a 1.5 T scanner (Siemens Sonata, Germany) equipped with 40 mT/m gradients and a standard circularly polarized head coil, using a blood oxygenation level-dependent response (BOLD) sensitive T2* weighted single shot gradient recalled echo (GRE) echo planar imaging (EPI) sequence (TE/TR 50/3000 ms) resulting in voxel dimensions of 3x3x3 mm^3^. 400 volumes of 35 slices each were thus acquired during both baseline and condition of interest. In the same scanning session we also recorded a T1-weighted magnetization prepared rapid acquisition gradient recalled echo series (MPRAGE; 1x1x1mm^3^; TE/TR 3.76/1700 ms) and a T1-weighted spin echo series (SE; 1x1x1,5 mm^3^; TE/TR 15/700).

### fMRI tasks

Each scanning session consisted of 5 repeated ‘blocks’ during which subjects were instructed to either rest, or to perform the PASAT or PVSAT. Subjects were presented a sequence of numbers. After each stimulus digit—starting with the second—subjects had to calculate the sum of the two last stimuli. Subjects were instructed to press a button with their dominant hand (all subjects were right-handed), if this sum was equal to or greater than 10. Both modalities (PASAT and PVSAT) were performed twice in each block. The first time at a rate of one stimulus per 3 seconds (either visual (V_3_) or auditory (A_3_)) and afterwards at a rate of once per 2 seconds (V_2_ and A_2_). Numbers appeared on screen for the duration of 1s at a visual angle of 12°[5]. Auditory stimulations were performed in a normal spoken rhythm. Each of the five consecutive blocks was therefore structured R-V_3_-R-V_2_-R-A_3_-R-A_2_, where R denotes 30 seconds of rest. During auditory tasks, subjects were instructed to fixate on a dot in the middle of the screen. During rest, subjects were instructed not to move their body, head or eyes and to fixate on a dot in the middle of the screen and to stop performing any mental calculations.

### Pre-processing

fMRI data was pre-processed using Statistical Parametric Mapping software (SPM12; www.fil.ion.ucl.ac.uk/spm), using the following steps: realignment, slice timing correction, affine coregistration with structural T1 images, segmentation, normalisation into the standard Montreal Neurological Institute (MNI) space, and spatial smoothing with a Gaussian kernel of 8 mm full width at half-maximum.

### Independent component analysis

We then applied independent component analysis (ICA) to divide the data into separate components, using the MIALAB GIFT toolbox (http://mialab.mrn.org/software/gift/version2.0a). ICA divides fMRI data into a pre-defined number of maximally independent components, producing temporally coherent brain networks. Each component has a spatial map and a unique activation time course. The number of components was estimated by GIFT to be 27, using minimum description length (MDL).

Components were then compared to a dataset, as made available online by Allen et al. (http://mialab.mrn.org/data/ [8]). This dataset contains 75 resting state components, most of which were labelled. We resliced this dataset to have the same dimensions as ours, using ‘affine coregistration’ from the SPM toolbox, and used a correlation measure to compare the two sets. We used the highest correlation values to identify and name our components in an automated way. We then inspected all components manually to verify the automated naming process. Components that did not acquire a label were excluded from the subsequent analysis.

We collapsed the data for statistical processing. One timeseries was available per subject and per component, consisting of 5 blocks of 10 samples for each difficulty. Per subject and per component, the 5 blocks were averaged out to one average block per difficulty. Then the average block of each difficulty was reduced to one value by taking the mean. As such we obtained a single, representative value for each level of difficulty, for every component, for every subject.

### Connectivity

Connectivity matrices between networks were estimated for each subject and during different task versions. Connectivity was defined as the correlation of the time course concatenation during the respective task versions. This was done between all component pairs.

### Statistics

We performed an Analysis Of Variance (ANOVA) twice for every component to compare the representative values of the different difficulties: once (A) using test difficulty as a single factor in a one-way ANOVA and a second ANOVA (B) with two factors: modality (Visual vs Auditory) and stimulus presentation frequency (3s vs 2s) in a two-way ANOVA. We then performed a similar ANOVA analysis, one- and two-way analyses on the correlation matrices. All reported p-values are FDR corrected.

The order of difficulty we used was based on previous behavioural data published by our group [5]: from highest difficulty to lowest: A_2_ > V_2_ > A_3_ > V_3_.

## Results

### Identification of ICA components

**Fig 1:**
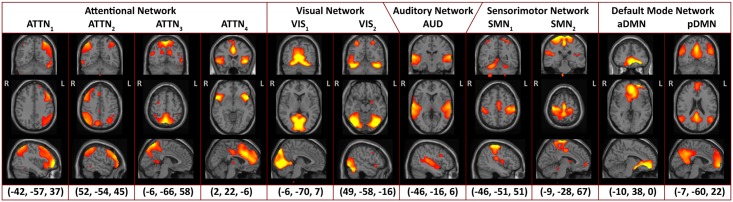
Spatial maps of the 11 labelled components at the three most informative slices. Coordinates are given in MNI space and images are shown in radiological convention (left is right).

**Fig 2:**
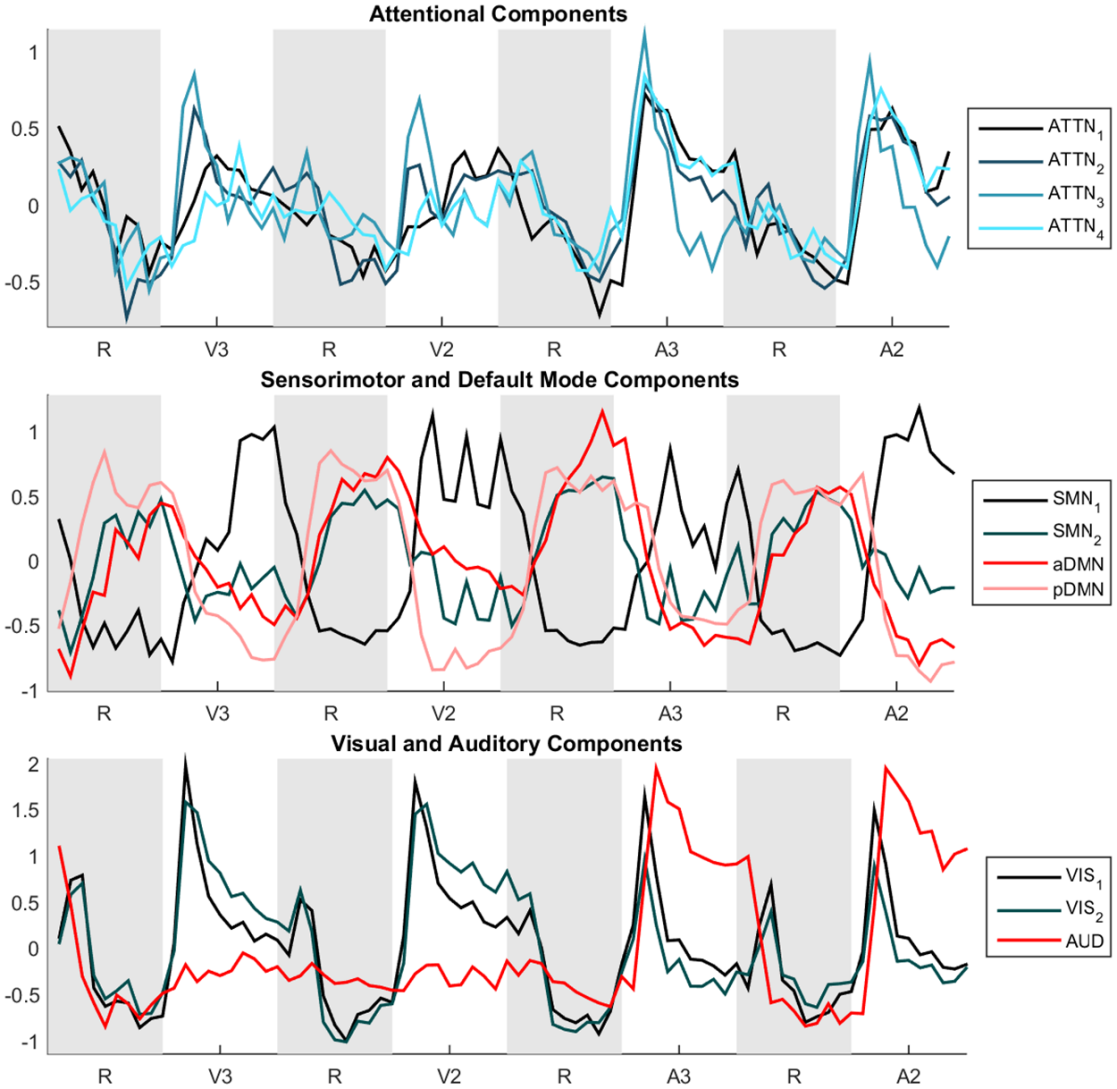
Component time courses, averaged over a window the length of one complete task block. (ATTN: Attentional, SMN: Sensorimotor Network, DMN: Default Mode Network, VIS: Visual, AUD: Auditory.)

We retrieved 11 labelled components from our automated labelling procedure (Table 1). Spatial maps and labels of the components are shown in Fig 1. Fig 2 shows time course activations, averaged over a window containing a full task block. Two components were identified as default mode networks (DMN), where pDMN forms a full map with more posteriorly located activations as described by Buckner *et al*. [9], and aDMN denotes an anterior DMN component. Two components were identified as being sensorimotor networks (SMN). SMN_1_ is a component containing the postcentral gyrus and the juxtapositional lobule cortex (a.k.a. the supplementary motor area). SMN_2_ is comparable to SMN_1_, but is found more anteriorly; with activations in the central opercular cortex, as well as the pre- and postcentral gyri. SMN_1_ attaches a greater weight of the left postcentral gyrus and is considered left-lateralised. The three highest correlations were with the following reference components: sensorimotor components 29 and 24 (R = 0.47 & R = 0.27) and auditory component 17 (R = 0.32). Furthermore, visual (VIS) and auditory (AUD) networks can be found in VIS_1_ + VIS_2_ and AUD respectively. VIS_1_ is a central, posterior component with activations in the lingual gyrus and the precuneus cortex; VIS_2_ has bilateral activations in the lateral occipital cortex and the fusiform gyrus. AUD describes a classic auditory network with activations in the superior temporal gyrus. Finally, four components form attentional networks (ATTN). ATTN_1_ and ATTN_2_ describe left and right frontoparietal attentional networks (FPN) respectively, similar to the ones found by Cruz-Gómez *et al*. [10], while ATTN_3_ activates the precuneus cortex and ATTN_4_ is an anterior cingulate and insular network.

**Table 1:** Extracted components with their corresponding component groups and numbers from the reference dataset (Allen et al [8]), and the correlation between the two.

Component	Allen #	Group	R
ATTN_1_	34	Attentional	0.42
ATTN_3_	72	Attentional	0.49
VIS_1_	64	Visual	0.61
ATTN_2_	60	Attentional	0.6
VIS_2_	39	Visual	0.52
aDMN	25	Default Mode	0.47
ATTN_4_	55	Attentional	0.61
SMN_1_	23	Sensorimotor	0.62
AUD	17	Auditory	0.62
pDMN	53	Default Mode	0.62
SMN_2_	29	Sensorimotor	0.47

### Amplitude analysis

**Fig 3:**
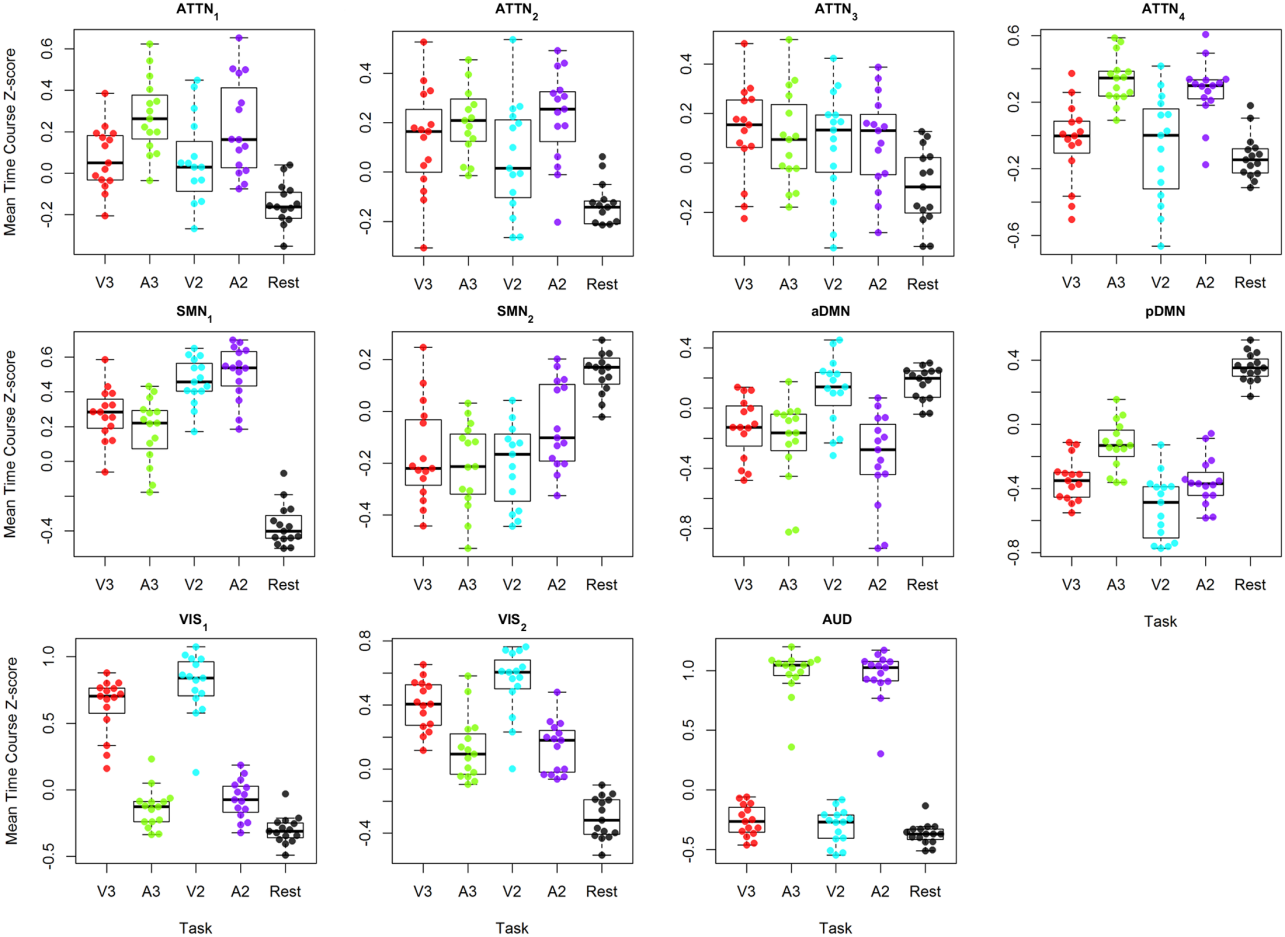
Box and scatter plots of the mean activity during the different tasks difficulties of all components. In a single component plot, mean activity during each part of the task block is shown for every subject. (ATTN: Attentional, SMN: Sensorimotor Network, DMN: Default Mode Network, VIS: Visual, AUD: Auditory.)

In Fig 3, the mean activity of the components is shown in box and scatter plots for the different paradigms (auditory/visual and 2s/3s delay), results of the ANOVA analyses are shown in Table 2.

**Table 2:** Results (as p-values) of ANOVA analyses (rightmost = interaction modality frequency), significant results are shaded red. The first 7 columns show the results of a 1-Way ANOVA (1WA) with difficulty as a factor, and comparisons between each difficulty pair. The last three columns show the results of 2-Way ANOVA with modality and frequency as factors, with the interaction term. All p-values are FDR corrected.

Component	1WA difficulty	1WA difficulty A3-A2	1WA difficulty V2-A2	1WA difficulty V3-A2	1WA difficulty V2-A3	1WA difficulty V3-A3	1WA difficulty V3-V2	2WA modality	2WA frequency	2WA modality:frequency
ATTN_1_	0.0142	0.9998	0.1845	0.3759	0.0291	0.0880	0.9998	0.0020	0.5917	0.9998
ATTN_2_	0.1527	0.9998	0.2311	0.8065	0.2112	0.7668	0.9836	0.0340	0.6810	0.7620
ATTN_3_	0.9998	0.9998	0.9998	0.9998	0.9998	0.9998	0.9998	0.9998	0.7886	0.9620
ATTN_4_	0.0000	0.9998	0.0031	0.0098	0.0003	0.0010	0.9998	0.0000	0.6242	0.9998
VIS_1_	0.0000	0.9998	0.0000	0.0046	0.0000	0.0030	0.2796	0.0000	0.2203	0.2973
VIS_2_	0.0000	0.9836	0.0000	0.0000	0.0000	0.0000	0.2203	0.0000	0.0571	0.5897
AUD	0.0000	0.9998	0.0000	0.0000	0.0000	0.0000	0.9998	0.0000	0.7063	0.9836
SMN_1_	0.0000	0.0000	0.9998	0.0025	0.0002	0.5917	0.0252	0.8065	0.0000	0.1845
SMN_2_	0.2612	0.2311	0.5535	0.5917	0.9998	0.9998	0.9998	0.6810	0.2398	0.2112
aDMN	0.0006	0.8597	0.0004	0.2742	0.0123	0.9922	0.1291	0.0007	0.5534	0.0261
pDMN	0.0000	0.0048	0.0938	0.9998	0.0000	0.0093	0.0571	0.0002	0.0001	0.7515

#### Attentional networks

Attentional networks ATTN_3_ and ATTN_2_ are not significantly influenced by task difficulty, with only ATTN_2_ having a slight effect of modality (p<0.05). The other two attentional components—ATTN_1_ and ATTN_4_—are significantly influenced by modality, and activation is more pronounced during auditory testing. In ATTN_1_ specifically, differences when modality but not frequency changes (V3 vs A3 and V2 vs A2), are not significant, only when comparing V2 to A3 (p<0.05), and this is not the case in ATTN_4_ where effects of modality only are apparent.

#### Auditory and Visual networks

As expected, these components clearly manifest an overall effect of modality and difficulty (p<0.0001), but not of stimulus frequency.

#### Sensorimotor components

The first sensorimotor component (SMN_1_) seems to be mediated by the applied delay–with higher activity in the shorter delay tasks, and task modality having no significant effect. The second sensorimotor component (SMN_2_) seems not to be significantly affected by any parameter change.

#### Default Mode Network components

As mentioned above, two components closely match with Default Mode Networks (DMN). These components deactivate during task performance and reactivate while subjects are idle. While the first of these DMN networks (aDMN) shows an inconsistent effect of task, V2 activations are significantly higher than during the auditory tasks A2 and A3 (p<0.001 and p<0.05 respectively), but not V3. pDMN, on the other hand, is clearly influenced by task modality (p<0.001); with higher activity during auditory conditions.

### Connectivity analysis

**Fig 4:**
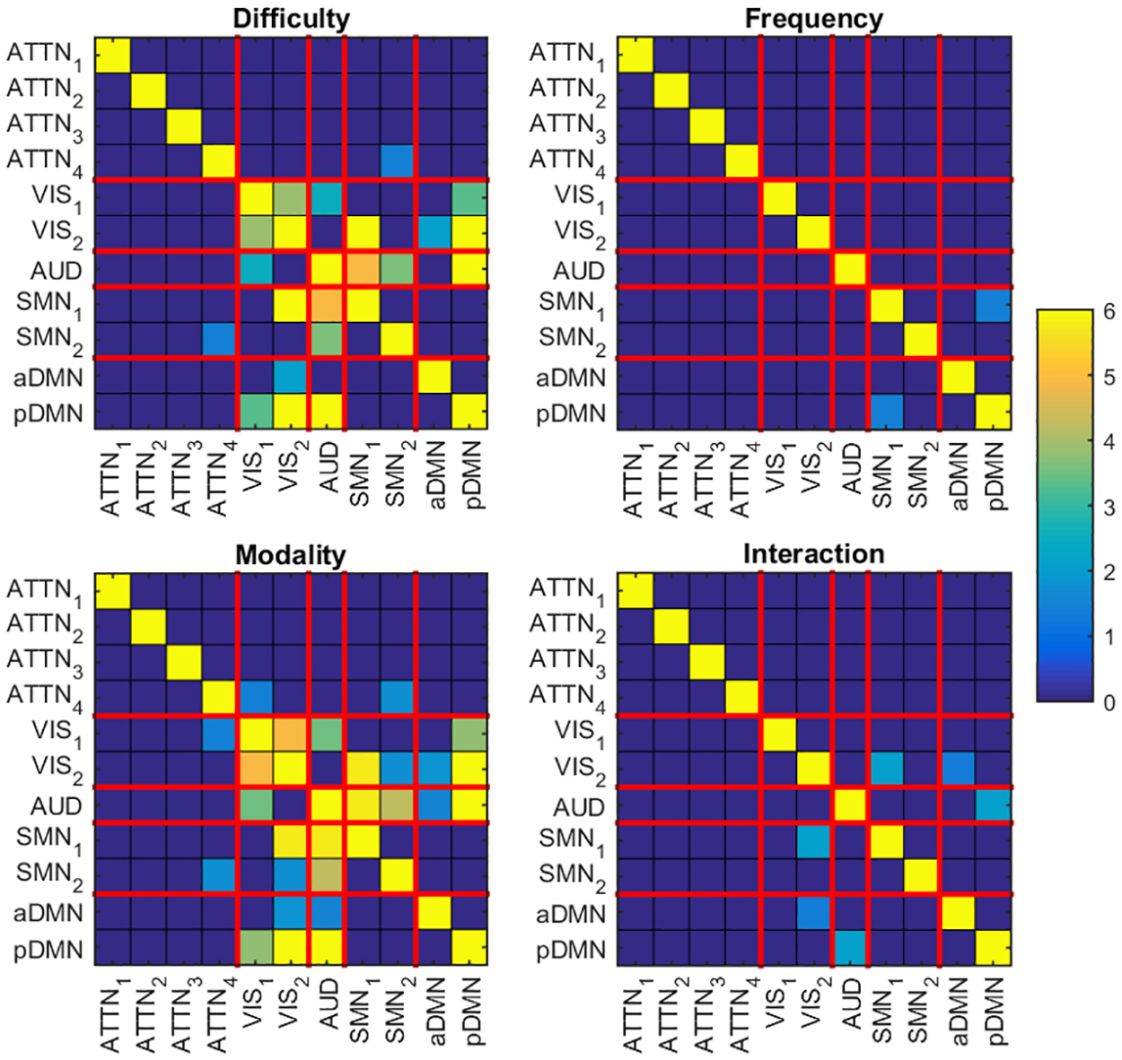
ANOVA results on component connectivity. Difficulty was based on a one-way ANOVA, modality and frequency were factors in a two-way ANOVA. Each voxel shows the -log(p) value of the respective component-pair connection. P-values of >0.05 are shaded purple. (ATTN: Attentional, SMN: Sensorimotor Network, DMN: Default Mode Network, VIS: Visual, AUD: Auditory.)

In Fig 4 the results of the ANOVA analyses are shown. Significant differences are shown in Table 3. In most cases, significant differences are found in edges linked to either visual or auditory components. This is to be expected in the case of the difficulty and modality based analyses. The edge linking ATTN_4_ with SMN_2_ experiences a significant effect of both difficulty and modality. A difference based on task frequency is found in the edge connecting SMN_1_ with pDMN. In both cases we see anti-correlation between the components, indicating that one component tends to deactivate as the other activates.

**Table 3:** Significant edge differences based on task difficulty, modality and frequency as well as the interaction term. All p-values are FDR corrected (NS: Not Significant).

Component 1	Component 2	Difficulty	Modality	Frequency	Interaction
SMN_2_	ATTN_4_	0.036	0.017	NS	NS
SMN_1_	pDMN	NS	NS	0.038327	NS
VIS_1_	ATTN_4_	NS	0.038	NS	NS
VIS_1_	VIS_2_	<0.001	<0.001	NS	NS
VIS_1_	AUD	0.0035	<0.001	NS	NS
VIS_1_	pDMN	<0.001	<0.001	NS	NS
VIS_2_	SMN_1_	<0.001	<0.001	NS	0.0075
VIS_2_	SMN_2_	NS	0.017	NS	NS
VIS_2_	aDMN	0.00698	0.016	NS	0.046
VIS_2_	pDMN	<0.001	<0.001	NS	NS
AUD	SMN_1_	<0.001	<0.001	NS	NS
AUD	SMN_2_	<0.001	<0.001	NS	NS
AUD	aDMN	NS	0.03	NS	NS
AUD	pDMN	<0.001	<0.001	NS	0.0075

## Discussion

In this paper we sought to investigate the effects of stimulus modality (auditory and visual) and frequency (every 3 seconds vs every 2 seconds) of a commonly used test to assess information processing speed and working memory in various clinical settings. Brain activation in different regions was analysed to assess potential differences.

### Task performance

fMRI scanning during PASAT task performance limits subjects’ response options: vocalisation of the answer would provoke head movement and cause susceptibility artefacts. Several solutions for this problem have been suggested—subjects can be asked to use a joystick [11] to indicate the right answer out of two options, or to raise a finger when the answer equals a certain predetermined number [12,13]. In this study, subjects had to press a button when their answer exceeded the value of 10. Subjects’ responses were not recorded. During scanning, a researcher was always present to make sure subjects were performing tasks correctly. Our experiment setup differs from previous studies as we did not include a control condition during visual testing nor instructed subjects to close their eyes during auditory testing [12,14]. We deemed these changing conditions to be too taxing for the subjects, yet this could lead to differing results.

### Amplitude analysis

#### Attentional networks

Modality based differences were found in three ATTN components, namely the left and right frontoparietal networks, designated as ATTN_1_ and ATTN_2_, as well as the anterior cingulate and insular network, named ATTN_4_.

Frontoparietal networks are important for attention, working memory and cognition [10,15,16]. These networks are utilised in multi-tasking behaviour and assist the encoding of visual, auditory, motor and rule information [17]. These networks (left and right) contain regions such as the Frontal Eye Field (FEF) and the Superior Parietal Lobule (SPL). Modality effects we found in the frontoparietal network partly confirm the findings of Tüdos *et al*, who describe higher activations in the right FEF during auditory tasks compared to visual tasks and suggested that the increased difficulty of auditory testing could be reflected in greater neuronal activation. Activation within the right frontoparietal network we described above shows significantly different activations between modalities, but not between difficulty levels.

The effect of modality was especially apparent in the anterior cingulate and insular attentional network ATTN_4_. This network is implicated in maintaining (complex cognitive) tasks and conflict processing [8]. Our results–namely the higher activation in ATTN_4_ during auditory testing—are compatible with results from recent publications. A similar component, too, had higher activations during a flanker task. This task consisted of the appearance flanker arrows which did NOT indicate the correct response [18]. This illustrates its involvement in maintaining intellectual effort, despite conflicting cues.

Increased activation during auditory tasks might be explained by the increased risk of error during PASAT, compared to PVSAT. Many studies have illustrated decreased performance on the former as opposed to the latter, in multiple groups of subjects [19]. This could provide an alternative explanation for the increased activity during auditory tasks in areas such as ATTN_4_. The anterior cingulate cortex especially has been implicated in the processing of ‘negative’ outcomes such as errors [20].

ATTN4, with its cingulate and insular components, seems to be influenced heavily indeed by unexpected and conflicting stimuli. We hypothesize, therefore, that it may be part of a salience network, reflecting the additional strikingness of auditory stimuli or new visual ones [21]. This would lend further support to the idea that these interventions and changes necessitate significant cognitive effort.

ATTN_3_ activity was not modulated by any parameter. This component mainly contains the precuneal nucleus and the lateral occipital cortex. The precuneus is implicated in mental imagery and episodic memory retrieval, as well as directing attention [22] and interacts with several brain networks [23]. Even though we notice activation during all task performances, our results do not show any noteworthy differences between task difficulty, nor stimulus modality or frequency. This possibly suggests some sort of ‘basic’ activation of the precuneus during these tests.

#### Sensorimotor components

Stimulus frequency-based differences were observed in SMN_1_. In SMN_1_ it was apparent that tasks with a 2s inter-stimulus delay induced higher activations than tasks with a 3s delay, independent of test modality. This may look surprising; as the networks’ name suggest an involvement mainly in processing sensory information and steering motor actions, yet there exists a role of sensorimotor areas in cognitive functioning [24]. The frequency effect might be the result of an increased amount of button presses. Areas included in SMN_1_ are the precentral gyrus, the central opercular cortex, and the juxtapositional lobule cortex. No significant effects were found for SMN_2_. The behaviour of this component was unexpected, as can be seen in Figs 1 & 2. From Fig 2 it is clear that this component exhibits task-negative behaviour. Nevertheless, it didn’t show any correlation with reference default mode networks (another task-negative network) and anatomically resembles the sensorimotor networks described in other publications [25,26]. Since all subjects were right-handed, the task-negative behaviour could also be explained by ipsilateral activation or contralateral deactivation. SMN_1_ has more active area on the left, with increased activation during higher frequency tasks, while SMN_2_ is slightly right lateralised.

#### Default Mode networks

Both DMN components experienced a significant effect of task modality, but only pDMN exhibited an effect of frequency. In pDMN—a traditional default mode network—the effect of frequency seems to be based on lesser deactivation during A3 when compared to all other series. As expected, DMN regions show decreased activation during task performance and increased activation during idleness [27]. The DMN has been shown to deactivate more during more complicated tasks [28]. This was also the case in our results–for example when we compared A3 to V2 and A2. Contradictorily, A2 does not cause more deactivation than V2, while V3 deactivates more than A3. While most task transitions followed a gradual increase of difficulty, V3 testing always followed either after A2 (a jump from most difficult to easiest) or was the first test in a session. This might have influenced deactivation during V3, with more difficulty being anticipated. If we separate DMN results by modality, it deactivates more during A2 than during the (easier) A3 task. While V2 tends to have lower activations than V3, this effect is not significant. The modality based differences in pDMN activation are clear, but contradictorily to what we might expect, visual tasks deactivate the DMN *more* than the auditory tasks—which are usually considered harder. Anderson *et al*. suggested that the DMN activates during response-irrelevant stimuli [29]. During PASAT, subjects were asked to fixate on a dot in the middle of the screen to reduce eye movements. We hypothesize that this may have caused visual distraction. We consider auditory distractions to have been less likely, or at least constant; such as the noise produced by the scanner. DMN deactivation could be influenced by stimulus duration: duration of visual stimuli was 1s, while auditory stimuli generally take 500ms on average [30]. Longer visual than auditory stimulation could increase DMN deactivation during visual tasks. Alternatively it has been shown that DMN deactivation decreases during repetitive encoding [31], so longer stimulation time could have the same effect, where DMN deactivation would instead decrease during visual stimulation.

#### Auditory and Visual networks

As expected, both Auditory and Visual components exhibited the effects of test modality. AUD contains the auditory cortex [32], with activations during auditory stimulation significantly overpowering those during visual testing or rest. In the occipital visual components, the opposite effect is clear, though deactivations during auditory testing are not as strong as one would expect, and spikes of activation appear in both components at every parameter change—even during rest. This might be explained by the subjects being instructed to focus on a dot on the screen at all times. Another possibility is that humans are visually inclined in general, and that a change of stimulation within the scanner could bring about some visual attentiveness and random saccades.

### Connectivity analysis

As expected, we see clusters of connectivity within the functional domains of ATTN, VIS and DMN. Negative correlations between the DMN components and all others, show how the DMN works in contrast to other brain regions that are active during attentive states and sensory processing [9].

Few differences exist between the networks: most of the edges that differ between tasks are connected to a visual or auditory edge. Considering the specialisations of these networks within their respective modality, it is no surprise that edges connected to them exhibit similar dependences.

Our results demonstrate that two component connections undergo significant changes during different difficulty levels. The connection between SMN_2_ and ATTN_4_ is stronger (in anti-correlation) during auditory tasks, while the connection strength between SMN_1_ and pDMN increases with higher stimulus frequency. SMN_2_ is shown to be a task-negative network and the interaction with ATTN_4_ might be explained by an increased top-down inhibitory influence on an idle part of SMN network. This suggests an increased communication between these components during harder tasks. Greater connectivity between default mode and attentional networks has been linked to a poorer ability of distractor suppression [33], which might explain why tasks are experienced as being more difficult.

### Weaknesses

Our sample size is limited (n = 15), and we only examined healthy, female controls in this study. Repeating the study with a larger group of volunteers and including patients with decreased cognitive function (e.g. MS patients) could bring to light bigger differences between the groups, and therefore generate a better understanding of functional brain network activity. While our subjects’ responses were observed during scanning for evaluation of protocol compliance, we did not save the results for further behavioural analysis. Different task difficulties were presented to subjects in a fixed order, our experiment could be improved by replication with shuffled task orders, to avoid a possible priming effect. Finally, we did not include motor control conditions to account for movement artefacts as perfomed by Rachbauer et al [14].

## Conclusion

We found modality-based differences in functional brain network activation during MRI-adjusted PASAT and PVSAT. The evidence for any difference between frequency-based test difficulty, however, is sparse.

We noticed a clear effect of modality switch on attentional and default mode networks, even though we have suppressed the interfering effects of response vocalisation. Stimulus frequency only affected the sensorimotor network.

We conclude that the PASAT and PVSAT tests should not be considered interchangeable, while difficulty level within one type of the test does not seem to matter a lot. This should be taken into account when designing clinical trials and neuropsychological studies that seek to explore cognitive status in patients and healthy controls.
